# Sigmoid Colon Migration of an Intrauterine Device

**DOI:** 10.1155/2014/207659

**Published:** 2014-07-22

**Authors:** Funda Akpinar, Esra Nur Ozgur, Saynur Yilmaz, Oguzhan Ustaoglu

**Affiliations:** ^1^Department of Obstetrics and Gynecology, Etlik Zubeyde Hanim Women's Health Training and Research Hospital, 06010 Ankara, Turkey; ^2^Department of Obstetrics and Gynecology, Rize State Hospital, 53100 Rize, Turkey; ^3^Department of General Surgery, Rize State Hospital, 53100 Rize, Turkey

## Abstract

*Background.* Intrauterine devices (IUD) are commonly used birth control methods. Colonic perforation is an infrequent but serious complication of IUD. *Case.* A 34-year-old woman with 2-years history of IUD, inserted at early puerperal period, presented to gynecologist with chronic pelvic pain and dyspareunia. Radiological assessment revealed that there were two copper-T devices: one in uterine cavity and another in the colonic lumen. Attempts of retrieval with colonoscopy and laparoscopy were unsuccessful. Intrauterine device embedded in sigmoid colon wall was removed with resection of the involved segment and primary anastomosis was performed. *Conclusion.* Although there are cases in literature that are successfully managed with colonoscopy, in chronic cases, formation of granulation tissue complicates retrieval of an IUD by this intervention.

## 1. Introduction

Intrauterine devices are effective, safe, and widely used birth control methods, accounting for 16.5% use in undeveloped countries and 9.4% use in developed countries [1]. The incidence of uterine perforation by IUD is reported to be between 1.3 and 1.6 per 1000 insertions [2], which is relatively infrequent but potentially a serious complication. Perforations of IUD may occur either immediately by improper insertion or years after insertion by device migration.

We report a case of an IUD penetrated to the wall of sigmoid colon that became symptomatic two years after insertion and discuss management of this situation.

## 2. Case Report

**Figure 1:**
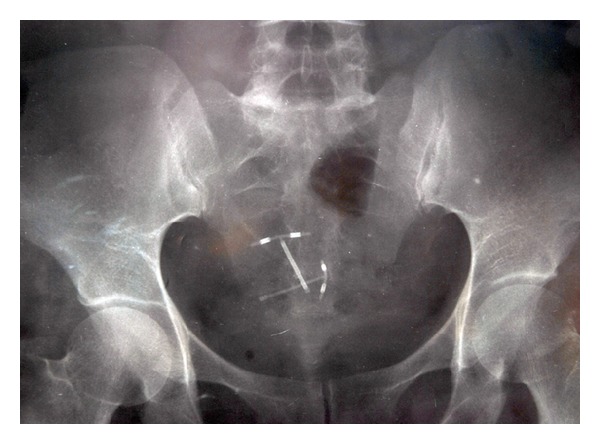
Plain pelvic X-ray showing two copper-T devices.

A 34-year-old woman presented with chronic pelvic pain, dyspareunia, and occasional episodes of bright red blood in her stools attributed to anal fissure. She had a history of a copper-T 380A IUD insertion at the sixth week of her puerperal period. Six months later, during routine control in the family planning center, IUD was not visualized and was thought to be expulsed with the postpartum bleeding and a new intrauterine copper-T device was inserted. Two years later, on admission to our clinic, she had normal vital signs and blood tests. Upon vaginal examination IUD strings (only one pair) were displayed. The transvaginal ultrasound revealed that a copper-T 380A IUD within the uterine cavity and another I-shaped echogenic focus were deeply embedded into the myometrium. An abdominal roentgenogram was performed that demonstrated two copper-T devices: one in the uterine cavity and another in the abdominal cavity or the colon (Figure 1). The abdominal computerized tomography scan confirmed that the IUD was located in the sigmoid colonic lumen.

**Figure 2:**
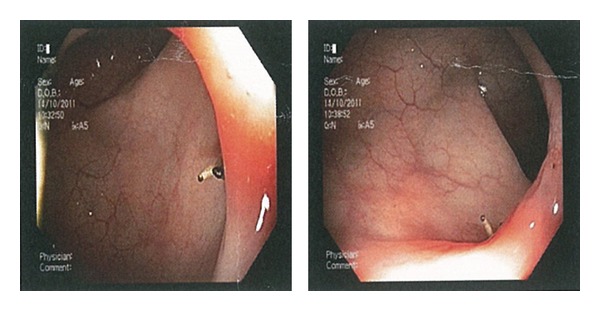
Colonoscopic view of the copper-T device partially penetrated to sigmoid colonic wall.

In an attempt to retrieve the device, colonoscopy was performed. At colonoscopy the arms of the copper-T device were seen in the lumen, but the coil wrapped body was firmly embedded into the wall of sigmoid colon (Figure 2). With the thought that retrieving by colonoscopy would be traumatic which results in an intraperitoneal leakage, we decided to carry out laparoscopy. Laparoscopy was unsuccessful due to the obliterated posterior uterine pouch. Following these findings the patient underwent laparotomy. The copper-T 380A IUD and associated granulation tissue were removed by resection of the involved area and primary anastomosis was performed. She was discharged on day two from the hospital with an uneventful postoperative period.

## 3. Discussion

Intrauterine device is a widely used reversible method of contraception, preferred due to long duration of birth control effect and ease of use. However it also has some serious complications such as perforation of the uterus and its migration to the abdominopelvic structures  [3]. The primary perforation may occur during insertion by mechanical forces. Some of the known risk factors for IUD perforation are inadequate training of family planning providers, insertion at early puerperal period when uterus is soft and bulky, past history of perforation (formation of a new canal with previous inappropriate insertion), and anatomically highly (ante- or retro-) flexed uterus. Secondary uterine perforation is also possible which is usually manifested by missing strings as in our case.

The symptoms of an IUD perforation are diverse varying from a subsequent unwanted pregnancy [4] to irritant lower urinary tract symptoms [5], chronic pelvic pain, peritonitis, and fistulae or abscess formation depending on the organ of penetration and the interval since penetration and the patient's response.

Ultrasonography and plain X-ray are diagnostic for echogenic and radio opaque foreign body, respectively. The computed tomography scan is a helpful imaging technique as in our case for confirmation of the localization of IUD.

World Health Organization recommended removal of a dislocated IUD as soon as possible irrespective of their type and location [6]. It is advised to retrieve a migrated IUD by minimally invasive techniques [7]. Endoscopic techniques such as colonoscopy, hysteroscopy, and cystoscopy can be used for diagnosis and treatment depending on the location of IUD. There are several case reports in the literature about removing an IUD in colonic lumen with colonoscopy. However retrieval of an IUD with colonoscopy when IUD is embedded in the colonic wall and surrounded with granulation tissue, as in our case, is inappropriate. This intervention would be traumatic and may cause colonic defect with intra-abdominal leakage of colonic content [2]. A review of surgical techniques to remove IUD revealed that 93% of the reported cases in literature attempted laparoscopically, but cases of both abdominal and pelvic organ perforations have the open surgery rate of 57.1% [7]. In our case, we attempt to retrieve the IUD firstly with colonoscopy then with laparoscopy. However full thickness perforation of the sigmoid colon, associated granulation tissue, and obliterated Douglas pouch that resulted in difficulty in access obligate us to laparotomy.

In conclusion although uterine perforation is rare, missing strings should be alerting about extrauterine placement of an IUD especially if it occurs in early postpartum insertion. Our experience suggests that removal of an IUD from full-thickness colonic wall perforation by colonoscopy could be traumatic.
